# 3D printed mask extenders as a supplement to isolation masks to relieve posterior auricular discomfort: an innovative 3D printing response to the COVID-19 pandemic

**DOI:** 10.1186/s41205-020-00080-7

**Published:** 2020-09-29

**Authors:** Zachary O’Connor, Daniel Huellewig, Peeti Sithiyopasakul, Jason A. Morris, Connie Gan, David H. Ballard

**Affiliations:** 1 3D Printing Center, Barnes Jewish Hospital, St. Louis, MO USA; 2 St. Louis Children’s Hospital, St. Louis, MO USA; 3 Student Technology Services 3D, Washington University, St. Louis, MO USA; 4 Washington University School of Medicine, St. Louis, MO USA; 5 Mallinckrodt Institute of Radiology, Washington University School of Medicine, 510 S. Kingshighway Blvd, Campus Box 8131, St. Louis, MO 63110 USA

## Abstract

**Purpose:**

Many commonly used mask designs are secured by elastic straps looping around the posterior auricular region. This constant pressure and friction against the skin may contribute to increased wearer pain, irritation, and discomfort. The purpose of this work is to report a modified 3D printed mask extender to alleviate discomfort and increase mask wearability by relieving posterior auricular pressure from isolation masks.

**Methods:**

Our institutional review board designated this project as non-human research and exempt. As part of resourcing 3D printing laboratories along with individual 3D printers to provide resources to healthcare workers, mask extenders were printed to relieve posterior auricular pressure from individuals wearing isolation masks. The authors modifed an existing mask extender, increasing its length with accompanying peripheral rungs for isolation mask securement. 3D printing was performed with Ultimaker S5 (Ultimaker B.V.; Geldermalsen, Netherlands) and CR-10 (Creality3D; Shenzhen, China) 3D printers using polylactic acid filaments. The author’s modified extended mask extenders were printed and freely delivered to healthcare workers (physicians, nurses, technologists, and other personnel) at the authors’ institution.

**Results:**

The final mask extender design was printed with the two 3D printers with a maximum 7 straps printed simultaneously on each 3D printer. Mean print times ranges from 105 min for the Ultimaker S5 printer and 150 min for the CR-10. Four hundred seventy-five mask extenders were delivered to healthcare workers at the authors’ institution, with the demand far exceeding the available supply.

**Conclusion:**

We offer a modification of a 3D printed mask extender design that decreases discomfort and increases the wearability of isolation mask designs with ear loops thought to relieve posterior auricular skin pressure and ability to control strap tension. The design is simple, produced with inexpensive material (polylactic acid), and have been well-received by healthcare providers at our institution.

## Introduction

The COVID-19 pandemic has upended many facets of healthcare provision across the world. The need to protect frontline physicians, nurses, technicians, and other staff from infection has produced one of the most visible changes: healthcare workers wearing masks, including respirators and isolation masks, for prolonged periods of time. In the setting of a pandemic, adherence to guidelines and infection prevention protocols is of particular importance, and the practical realities of following those instructions must be carefully considered to maximize compliance [1, 2].

A 2012 study showed that mask discomfort due to heat, pressure, and pain significantly increases with increased duration of wear, and that discomfort may influence compliance with protocols for personal protective equipment [1]. A survey of healthcare workers in China during the COVID-19 pandemic showed that 22% (90/404) of participants reported discomfort specifically due to the mask straps, more than any other source of discomfort [1]. At first consideration, this discomfort may seem trivial in comparison to the risks of the pandemic, but this discomfort has been associated with a unwillingness by health care providers to wear isolation masks for the duration of an 8 h shift even with scheduled breaks [3]. This discomfort may potentially decrease compliance with institutional guidelines even among a population of highly educated and motivated providers.

A common source of this discomfort is due to prolonged contact and friction between sensitive skin and the straps of the masks. Many commonly used mask designs are secured by elastic straps looping around the posterior auricular region. This constant pressure and friction against the skin may contribute to increased wearer pain, irritation, and discomfort. The purpose of this work is to report a modified 3D printed mask extender to alleviate discomfort and increase mask wearability by relieving posterior auricular pressure from isolation masks.

## Materials and methods

**Fig. 1:**
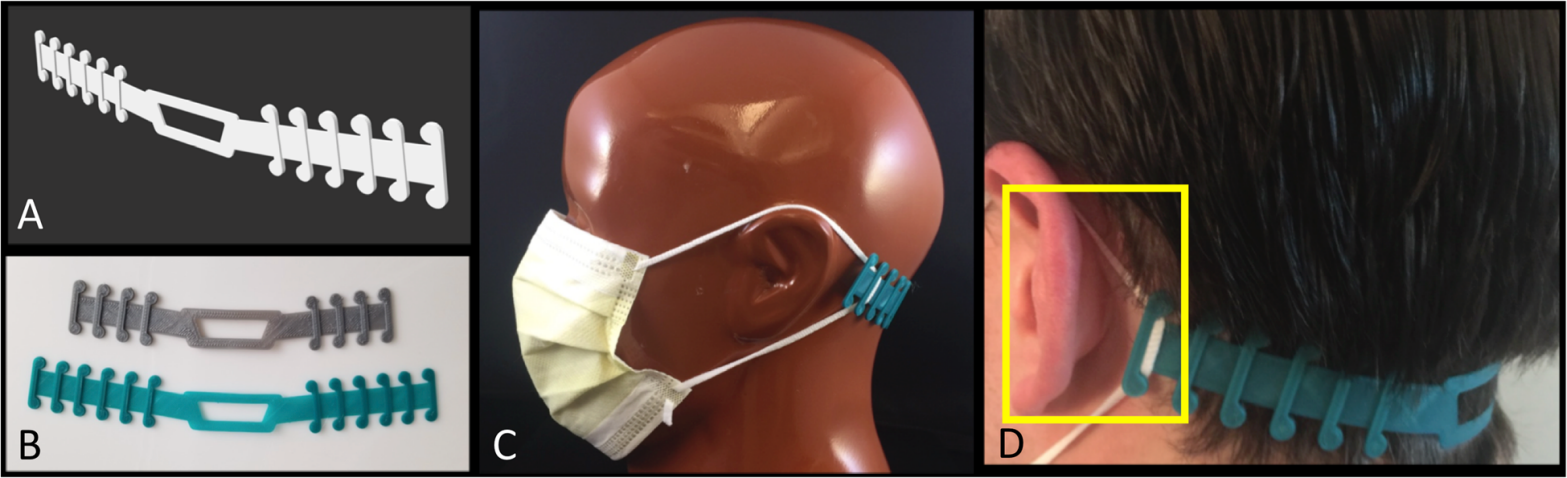
**a**. Stereolithography image file of the authors’ modified mask extender design. **b**. Photograph of 3D printed mask extender from the NIH 3D Print Exchange [4] (top, shorter design) and the authors’ modified and extended design (bottom, longer design). **c** and **d**. Photographs of the lengthened mask extender on a head mannequin (**c**) and healthcare professional (**d**). Note that the extender holds the mask ear loop off of the posterior auricular surface (yellow box in **d**)

**Fig. 2:**
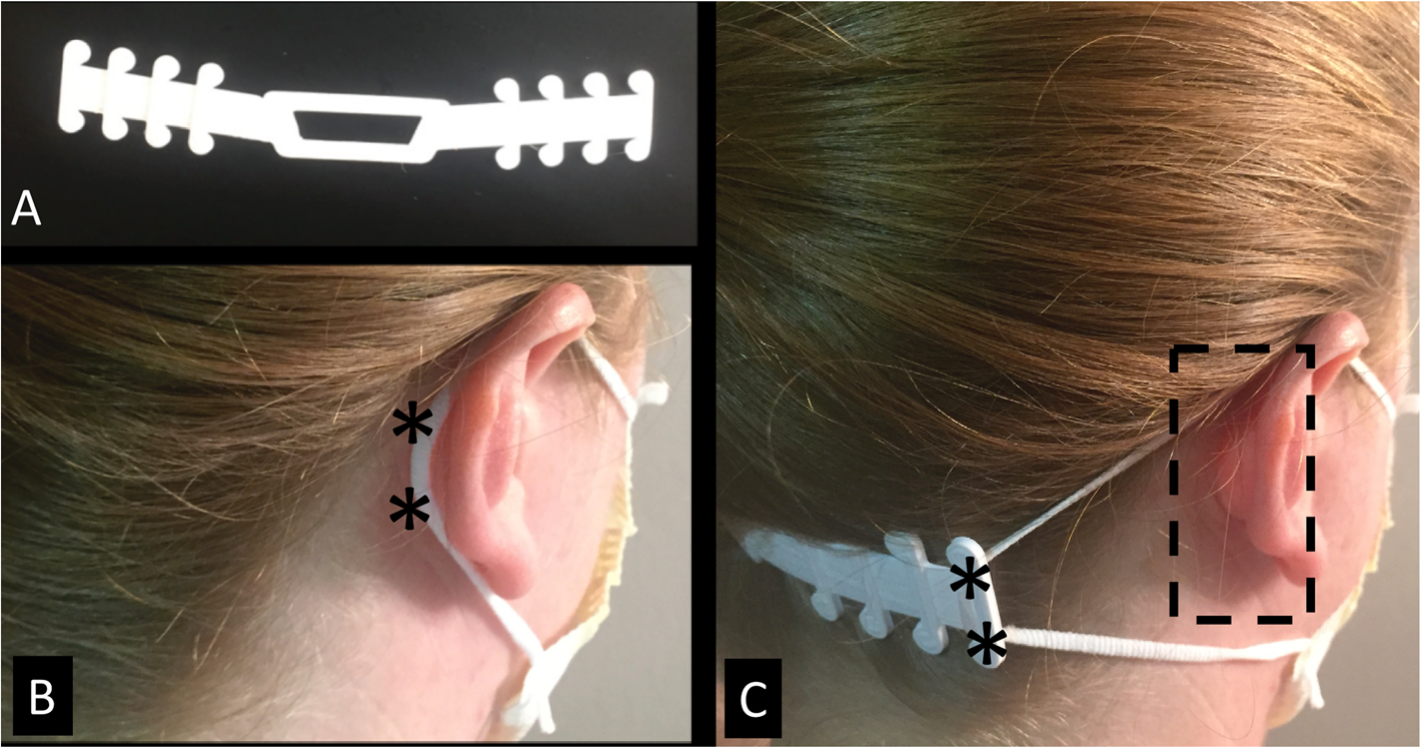
**a**. 3D printed mask extender from the NIH 3D Print Exchange open source design files [4]. **b**. Medical mask in its normal position with the strap resting behind the ear (strap position delineated by * *). **c**. Application of the 3D printed mask extenders allows the mask straps (* *) to be positioned off the back of the ear (dashed box)

Our institutional review board designated this project as non-human research and exempt and took place during the early COVID-19 pandemic [2], March 15 to May 212,020. As part of resourcing 3D printing laboratories along with individual 3D printers to provide resources to healthcare workers, mask extenders were printed to relieve posterior auricular pressure from individuals wearing isolation masks. The 3D printed mask extenders work with medical surgical masks (also known as medical masks and surgical masks) with elastic straps secured to the back of individual’s ears. A freely available online template was used initially [4] . This particular mask extender template was chosen because it was the only design on the NIH 3D Print Exchange reviewed for clinical use. Based on initial feedback the size was too small for some individuals. One of the authors (ZO), experienced in computer design, 3D modeling, and 3D printing technology (1 year of experience), modified the mask extender design (Fig. 1). An example of the modified design is presented in Fig. 1 while the original design is shown in Fig. 2. This design is greater in length with accompanying peripheral rungs for isolation mask securement and accounts for larger cranial sizes and individuals with larger volumes of hair. The author’s modification is available in the supplemental material (Supplementary Materials STL files 1 and 2). The slicing software used was Ultimaker Cura. 3D printing was performed with Ultimaker S5 (Ultimaker B.V.; Geldermalsen, Netherlands) and CR-10 (Creality3D; Shenzhen, China) 3D printers using polylactic acid (PLA) filaments. The print settings utilized were: i) an Infill of 20%, ii) print speed of 60 mm/s for Ultimaker 5 s and 55 mm/s for the CR10, iii) PLA temperature settings of nozzle at 215 °C for first layer, 210 °C for the rest and a temperature setting of 64 °C for the build plate (note should be made that the temperature range can change for the nozzle based on the filament color and manufacturer). No support or raft was required and the modified design and printing setup was optimized to print a high volume of extenders in the shortest time using the least possible materials. At completion of printing, the extenders were sprayed with disinfectant spray. The authors’ modified extended mask extenders were printed and freely delivered to healthcare workers (physicians, nurses, technologists, and other personnel) at the authors’ institution.

## Results

The final mask extender design was printed with the two 3D printers with a maximum 7 straps printed simultaneously on each 3D printer. Mean print times ranges from 105 min for the Ultimaker S5 printer and 150 min for the CR-10. At writing, 475 mask extenders have been printed, each delivered in batches of 50 extenders with requests for continued production. Through providers were not formally surveyed, direct feedback has been overwhelmingly positive. Feedback has noted that the expended life of the extenders is approximately 2 months with wear from reuse and the extenders are amenable for disinfectant wipes and sprays. The demand far outweighs the supply and rate they are able to be produced.

## Discussion

Although isolation masks are associated with less discomfort compared to N95 respirators, a prior study demonstrated that healthcare providers’ discomfort increases with prolonged use (greater than 2 h) of wearing isolation-type masks with ear loops and that the discomfort continues to increase on a per-hour basis [3]. The mask extender design allows for isolation masks to be secured behind the posterior aspect of the pinna without exerting direct pressure on it, diffusing that pressure broadly across the posterior aspect of the head. This design, first posted online to the National Institutes of Health 3D Printing Exchange [4], has been modified by the authors by lengthening it and adding additional rungs to increase comfort and increased ability to adjust the tension of the straps to achieve a more individualized fit. The combined relief of pressure with tension control decreases discomfort and irritation from mask designs with ear loops. As mask utilization continues to increase among healthcare providers in every setting and among the lay public, this is especially relevant as increasing wearability and decreasing discomfort may increase compliance with the infection control and public health guidelines put forth by local and national institutions.

The modified 3D printed mask extender in the current study was based on a design submitted to the NIH 3D Print Exchange by Davis Becker, an innovation specialist with the VHA Innovation Ecosystem [4]. The original design was created from Quinn Callander, a 13-year old boy scout from Maple Ridge, British Columbia [5]. That deign was then modified by Ken Lord [6]. From feedback from the frontline staff of the mask extender design, one of the authors (ZO) modified to extend the length and add extra rungs to account for the larger cranial sizes and staff with larger volumes of hair.

This design is part of a larger movement of community generated 3D printed solutions to novel issues and supply chain shortages that arose with the advent of this pandemic including ventilator components, personal protective equipment such as splash-proof face shields, surgical masks, N95 masks, N90 masks, powered air-purifying respirator hoods, and controlled air purifying respirator hoods, and environmental solutions such as door handle modifications [7]. 3D printing has attempted to solve this issue by facilitating manufacturing of ad hoc personal protective equipment as well as medical equipment during the COVID-19 pandemic [8–10]. These efforts have included producing face shields [8], innovative ventilator solutions [9], among others. The FDA have issued caution with 3D printed personal protective equipment [10], but they have expressed willingness to work with individuals and entities producing such alternatives and are currently working with the NIH 3D print exchange [10].

Limitations to this work include obtaining formal survey data from wearers and comparing user comfort or discomfort levels compared to not wearing a mask extender. Similar interventions in the 3D printing of face shields for interventional radiologists have been thoroughly evaluated and found to not produce any detriment to ability of physicians to perform their duties while solving the supply chain shortage of personal protective equipment [11]. Alternative materials can be used to 3D print these mask extenders. While we used PLA a semi-rigid to rigid polymer, polyethylene terephthalate glycol (PETG) is a more flexible material that is likely well-suited for the extenders; intended purposes. While we considered and explored using PETG polymer, at the time of the study (early COVID-19 pandemic - mid March to late May 2020), PETG was in limited supply and being used at our institution for 3D printed face shields. Furthermore, PETG requires higher print temperature settings for the nozzle and build plate, resulting in slower print speeds and overall longer printer times. These factors may not be optimal for mass production using few desktop 3D printers. For the mask extender design, the anecdotal support and metrics of satisfaction, including personal feedback and requests to produce more mask extenders, have been overwhelmingly positive, and the authors of this study thought it appropriate to share these results to offer this solution to a broader audience as well as encourage continued utilization and modification of 3D printed solutions as the world continues to grapple with the COVID-19 pandemic. Potential improvements to the 3D printed mask extender in the current study may be reducing the number of rungs and decreasing the material volume for faster print speeds and lighter weight of the extender.

In conclusion, the authors offer a modification of a 3D printed mask extender design that decreases discomfort and increases the wearability of isolation mask designs with ear loops thought to relieve posterior auricular skin pressure and ability to control strap tension. The design is simple, produced with inexpensive material (polylactic acid), and have been well-received by healthcare providers at our institution. The authors offer their design, which others may adapt.

## Supplementary information

**Additional file 1.**

**Additional file 2.**

## Data Availability

The datasets during and/or analyzed during the current study are available from the corresponding author on reasonable request.

## Abbreviations

NIH: National Institutes of Health
FDA: Food and Drug Administration
PLA: Polylactic Acid
PETG: Polyethylene Terephthalate Glycol
